# Green Innovation Behavior Toward Sustainable Tourism Development: A Dual Mediation Model

**DOI:** 10.3389/fpsyg.2022.930973

**Published:** 2022-06-10

**Authors:** XiaoJuan Li

**Affiliations:** Department of Business, Shandong Management University, Jinan, China

**Keywords:** green innovation, sustainable development, tourism, environmental CSR perception, attitude toward environmental CSR

## Abstract

The rapid increase in globalization has fostered the emerging ecological challenges to halt human civilization, substantially highlighting the need for environmental management. The study’s primary objective is to analyze the impact of environmental perception of corporate social responsibility (CSR) and attitude toward environmental CSR on sustainable tourism development while considering the mediating role of national park identification goals and employee pro-environmental behavior. The data was collected from the 338 employees working in the Chinese tourist firms’. The study variable’s reliability and validity was checked by using Composite Reliability (CR) and Average Variance Extracted (AVE). Study results show that environmental CSR perception and attitude toward environmental CSR positively impact sustainable tourism development, national park Goal identification, and employee pro-environmental behavior. National Park goal identification and employee pro-environmental behavior mediate between sustainable tourism development and environmental perception of corporate social responsibility and attitude toward environmental CSR.

## Introduction

In recent years, the fast-growing anthropogenic emissions (e.g., GHGs, air pollutants, intoxicants, and impurities) have elevated this meteorological phenomenon to widely deform the worldwide human heritage. In particular, this increased irradiation has irrefutably caused global climate change to erode the weather pattern, substantially damaging the natural legacy. Unfortunately, this escalated ecological diversity has pressed the world’s sustainability development (Wang et al., 2020), thus causing countries to form positive coalitions for addressing environmental concerns.

However, linking the environmental concerns to human welfare demands the sustainability model to maintain enduring performance (e.g., social, economic, and ecological), potentially ensuring nature’s well-being. Undoubtedly, today, sustainability has become a radical vehicle driving the company’s performance against the accelerating environmental challenges. Sustainability development reduces the adversity of the changing climate, thereby strengthening the ecological performance of universal sectors (Leung et al., 2019). Given the statement, the sustainability concept has taken a unique path for promoting the socio-ecological growth of global industries (e.g., tourism) (Higgins-Desbiolles et al., 2019).

The world’s tourism sector brings colossal economic benefits to the global nations. Unfortunately, besides immense financial advantages, the increasing environmental cost of tourism activities has made the industry experience an undeniable influence of universal climate change. Its extended broadness has massively damaged the world’s ecosystem, thus causing the industry to experience environmental crises (Scott et al., 2019). Based on this notion, this current situation raises questions about the industry’s sustainable development, thus prompting a need for sustainability action (Blázquez-Salom et al., 2019; Ajaz et al., 2020). Therefore, the literature suggests taking a deep look into the environmental emergencies (Hughes and Scheyvens, 2018), thereby adopting sustainability approaches to ensure the industry’s long-term survival.

Notably, this concept of sustainable development has become an emerging issue influencing the leisure industry. As a result, the increasing environmental deterioration deeply rooted in the tourism atmosphere has emphasized the need to develop a positive CSR perception. CSR is a vital topic in tourism, mitigating the impact of ecological damages (Han et al., 2020). Environmental CSR refers to the firms’ policies for achieving sustainability development (Aguinis, 2011; Shehzad et al., 2020). Based on this statement, the study shows that this profound CSR phenomenon has made employees adopt sustainability activities, thus ensuring environmental well-being (Abdullah et al., 2020; Afsar and Umrani, 2020).

Consistently, over the last few years, CSR has remained a leading focus of meteorologists for ensuring environmental sustainability. CSR achieving positive ecological influence demands employees to focus on sustainable practices (George et al., 2020). However, this increase in individuals’ capacity has made the employees develop a positive CSR attitude, thus realizing their responsibility toward sustainability practices. Given the articulation, the study shows that employees’ positive attitude toward CSR fosters their commitment to maintaining sustainability development (Fung Wong and Kim, 2020). Indeed, this employee’s environmental attitude alters the natural context. The CSR attitude encourages the employees to preserve the natural environment. This developing employee attitude toward natural protection makes the employees behave favorably toward the environment. Based on this statement, the study indicates that the employee’s positive environmental attitude addresses the ecological problems, thus making the employees exhibit ecological conversation behavior (Sarfraz et al., 2020; Gidlöf et al., 2021).

Employee pro-environmental behavior is a phenomenon that rationalizes the construct of environmental protection. In this context, the CSR perceptive enhances the organization’s ecological orientation by predicting positive environmental behavior (Islam et al., 2019). However, today, individuals and businesses controlling the growing vulnerability have engaged in pro-environmental behavior. Accordingly, academics establishing a link between the CSR practices and employees’ proactive behavior states that employee’s pro-active behavior helps the organizations deal with the increasing environmental challenges (i.e., climate change, atmospheric degradation) (Rezapouraghdam et al., 2018). Indeed, ecological behavior establishes a healthy environment by leading employees’ eco-friendly actions to achieve sustainable goals (Sarfraz et al., 2019; Halder et al., 2020).

Undoubtedly, with the increased popularity of CSR, tourism sites have faced a sustainability challenge, indicating the need for tourism protection. However, this extreme connectedness to the natural world demands that individuals protect their natural habitat against environmental vulnerability. National parks form a significant part in strengthening the nation’s environment. As a result, addressing these issues has become critical for protecting the country’s beauty. Natural parks massively contribute to the nation’s beauty by enhancing the quality of the local destinations. As a result, nature-based tourism has significantly gained growth worldwide by ensuring the development of outdoor activities. The national landscape requires proper management with firms’ recreational activities complementing the natural landscape conservation endeavor to improve the environmental conditions. In this regard, the CSR sustainable perception encourages the employees to enhance the beauty of the national parks. The CSR socio-environmental perceptive raise the tourism workers’ concern for land protection and sustainable goals (Lin and Lee, 2022; Sarfraz et al., 2022b).

Therefore, the above literature highlighting the research gap calls for an urgent need to explore the tourism industry concerning the sustainability frame. Sustainability development in the prior literature has been considerably ignored for several years, colossally affecting various national destinations (Cotterell et al., 2019). Given the illustration, the research shows that various tourist destinations have massively been damaged because of irresponsible human behaviors that urgently need correction (Wang et al., 2019). This expressed disclosure makes the researchers take a deep look into tourism sustainable practices. Moreover, the prior studies show that understanding the influence of environmental degradation has encouraged the employees to exhibit pro-environment behavior that has limitedly gained attention in the tourism industry (Latif and Aziz, 2018; Sarfraz et al., 2022a). Notably, this neglected area of research has harmed the environmentalist expectation to maintain environmental well-being. Therefore, the research gap demands investigation in the sustainability domain for preserving sustainable tourism development.

Significantly, in addition, to the need for considerable discussion on environmental sustainability development, this paper fundamentally highlights the prime drivers, minimizing the negative impact of the ecological degradation in the tourism industry. Against the limitations recognized in the prior literature, this study extends the knowledge on the environmentally friendly initiative (i.e., CSR) and sustainable tourism development. Furthermore, it also discusses the link between the employee attitude toward environmental CSR and sustainability tourism development. In the same vein, the study introduces the concept of the national park goal identification and employee pro-environmental behavior as a novel construct, mediating the relationship between the variables (e.g., CSR perception, attitude toward CSR perception, and sustainable tourism development).

Accordingly, the paper presents a multi-level sustainability model by colossally discussing the environmentally sustainable factors in the light of social and environmental psychology. This novel study highlights the key factors influencing sustainable tourism development. It extends the literature on the variables that need urgent investigation. Indeed, this is a unique consideration integrating the complex phenomenon (e.g., national park goal identification) from the nature-based tourism perspective. Significantly, the study’s findings provide new insight into different variables nurturing the employees’ pro-environmentally actions. The study outcomes suggest that researchers, practitioners, managers, and governments improve tourism performance by enhancing employees’ CSR psychology (i.e., perception, attitude, and behavior).

The study follows the following structure. Section 2 discusses the study’s theoretical background, while section 3 frames the research methodology. In the same vein, section 4 presents the study analysis, with section 5 discusses the study outcomes against the previous reviews. Lastly, section 6 concludes the research topic by demonstrating the study’s limitations and recommendations.

## Literature Review

### Environmental Corporate Social Responsibility Perception and Sustainable Tourism Development

Over the past decade, the increasing climate change has topped the world with massive environmental challenges. Particularly, with the increased human civilization, these problems have become the global concerns affecting tourism activities. In particular, the colossal depletion of natural resources and the rapid climate change have made the tourism division lack the sustainability model (Scott et al., 2019), thereby demanding the need for environmental CSR. In particular, CSR is a phenomenon that incorporates the ecological aspects into achieving societal welfare. In the tourism sector, corporate social responsibility elevates the need for implementing environmentally friendly initiatives, thus ensuring socio-ecological development (Paskova and Zelenka, 2019).

Indeed, enhancing the firms’ ecological growth by allowing the employees to adopt CSR activities is a few examples of the increased CSR popularity and commitment. Employees are solely responsible for addressing corporate environmental concerns. Given the articulation, the study shows that employees’ promising CSR initiatives support the increasing trend of sustainable tourism (Hassan et al., 2020). Accordingly, firms’ socio-ecological responsibility plays a critical role in sustainable development. Corporate social responsibility has a massive effect on the firms’ stakeholders (e.g., employees, government, and customers). Given the explanation, the study shows that the new paradigm of ecological responsibility in the tourism sector has shifted the focus on sustainable practices, thereby improving the country’s natural environment (Fang et al., 2018).

Undoubtedly, the employees’ CSR activities play a critical role in establishing an individual’s connection with the environment. It makes the employees adopt the tool as the rational justification for doing good. In explaining this notion, the research shows that the perceived CSR capability widely influences the employees’ perception, thus significantly enhancing sustainable tourism development (Boroujeni et al., 2021). Consequently, based on this literature, we have developed the following hypothesis:

*H1: Environmental CSR perception has a positive and significant impact on sustainable tourism development.*

### Environmental Corporate Social Responsibility Perception and National Park Goal Identification

National parks are the prominent tourist places that have widely located worldwide. Most importantly, it offers a wide range of diverse landscapes (e.g., natural habitats). Its natural attractions make individuals feel connected to its history, thus raising their concern for its preservation. The national parks hold unimpaired natural values that extend the benefit of outdoor recreational activities. However, despite its increasing significance, today’s parks have been considerably subjected to atmospheric distortion. This increased environmental influence has immensely halted the tourism sector, demanding the need for safeguarding biodiversity for future use. Given the articulation, the study shows that the significance of preserving the national park has increased in the last few years (Komossa et al., 2020).

In particular, today, the management objective is to safeguard the natural landscape by confirming the national parks as the gateway to boosting the country’s tourism activities. National parks are the areas that need protection for future generations. As a result, today, environmental management calls for devising preservation strategies for land protection and tourism enhancement. In recent years, the management activities have dealt the parks to face considerable ecological challenges, thereby elevating the need for modifying the stakeholder priorities regarding the land preservation. The CSR perception capitalizes on the stakeholders to manage the wildlife, local communities, and tourist places. Given the statement, the study shows that the increased CSR perception has made the employees understand their socio-environmental responsibilities for addressing the growing environmental challenge damaging the nation’s landscape (i.e., parks) (Sethi et al., 2020).

In particular, today, employees’ CSR initiatives have helped the organizations restore the world’s ecosystem for the future generation. Employees play a critical role in fostering the conditions of the national park. The employee CSR perspective makes them safeguard the earth’s landmarks for future visitors (Brankov et al., 2019). Indeed, the individuals’ love for the motherland makes them develop a positive CSR perception regarding organizations’ policies. Given the illustration, the study states that employees’ CSR perception maximizes organizations’ outdoor recreation activities, thus understanding the effect of increasing CSR on the environment’s well-being (Yılmaz and Anasori, 2022).

Therefore, this inception of the national park confirms that employees’ CSR identification improves the organizational prestige status and attractiveness. In particular, the employee CSR perception helps revitalize the earth’s beauty by persevering the natural areas (i.e., local heritage). However, the employees’ bonding with the natural environment plays a significant role in establishing a positive attitude toward the place’s identity. Based on this statement, the research shows that CSR bonding makes employees adopt pollutant-free activities, thereby ensuring environmental protection (Marasinghe et al., 2021). Based on these studies, the prior findings conclude the following hypothesis:

*H2: Environmental CSR perception has a positive and significant impact on national park goal identification.*

### Environmental Corporate Social Responsibility Perception and Employee Pro-environmental Behavior

Significantly, in recent years, the active organizations’ efforts addressing the environmental concerns have made employees act responsively toward the increasing ecological degradation. Accordingly, as the world is accelerating toward industrialization, the progressing environmental issues are colossally demanding CSR practices to strengthen individuals’ eco-friendly behavior. Based on this statement, the study shows that today, organizations are considerably motivating the employees to adopt socio-ecological activities, thus engaging them in pro-environment behavior (Cheema et al., 2020).

Employees’ pro-active behavior manifests in their CSR perception. Employees’ eco-friendly behavior encourages them to develop a positive CSR perception, thus combating the increasing negativity of global climate change. However, employees valuing nature endorse a serious environmental responsibility toward CSR practices. In explaining this notion, the research states that CSR, being a contextual factor, drives employees’ eco-friendly behavior to produce positive ecological outcomes (Ruepert et al., 2017). An individual’s environmentally friendly action promotes sustainable resource protection. It ensures the environment’s stability by confronting the growing climatic challenges. Concerning the tourism sector, the study indicates that the improved CSR perception has become a vital factor in accelerating employee environmental behavior (Tuan, 2018). As a result, many tourism organizations have promoted corporate social responsibility endeavors toward achieving eco-friendly tourism development (Han et al., 2020).

Hence, today, employees’ positive behavior has become the prime concern of meteorologists. The increasing ecological externalities have made corporate social responsibility initiatives trigger employees’ environmentally friendly behavior. Accordingly, the literature concludes that employees’ CSR perception inspires them to exhibit environmentally friendly behavior (Ture and Ganesh, 2018), thus establishing environmental wellness. Hence, the literature develops the hypothesis as follows:

*H3: Environmental CSR perception has a positive and significant impact on employee pro-environmental behavior.*

### Attitude Toward Environmental Corporate Social Responsibility, Sustainable Tourism Development, and National Park Goal Identification

Over the years, the environment laying the foundation of human survival has significantly made researchers focus on sustainable development. Perhaps, to understand this phenomenon, humans are continuously striving hard to reach ecological standards. Concerning this notion, today, the tourism industry is at a pivotal point, demanding the need for meteorologists’ attention. However, besides providing numerous development opportunities, the tourism sector is still combating the increasing ecological threat (Scott et al., 2019). In particular, the diverse forms of environmental challenges pose a profound risk to nature’s well-being, thus increasing the demand for environmental sustainability in the tourism sector.

Indeed, as an immediate response, the industry has called for shifting the focus toward sustainable tourism. Stakeholders play a vital role in embarking on holistic sustainability. The global significance of tourism development has made the employees realize their environmental responsibility. Employees’ positive attitudes stimulate their ecological responsibility. Given the explanation, the study shows that the employees’ dominant CSR perspective makes them develop a positive attitude toward firms’ sustainability practices (Wang, 2018). Moreover, the research reveals that firms’ positive attitude forms a critical antecedent in influencing firms’ sustainability practices toward achieving environmental protection (Cosma et al., 2021).

Hence, the rapid climate change has endangered humans to encounter massive calamities and disasters, thereby promoting the need for pro-environmental behavior. The increased environmental volatility has emphasized the need for environmentally friendly practices, thus ensuring the country’s ecological growth. However, the CSR initiative forms a valuable source of developing employees’ sustainable attitudes. The employee CSR perspective is the prime driver accelerating the individuals’ attitude and behavior to enhance the environmental conditions. Given the statement, the research shows that CSR activities alter the employees’ attitudes, thus exhibiting eco-friendly behavior (Su et al., 2018).

Perhaps, CSR activities profoundly alter the stakeholders’ attitude, substantially valuing the organizations’ functions. The employees’ attitude toward environmental CSR significantly encourages the individuals to exhibit environmentally friendly behavior. In particular, the employee valuing the pro-environmental activities engage themselves in organic activities, thereby influencing the individuals’ behavioral intention. Given the articulation, the study shows that employees appraising the CSR activities impute the organization’s beliefs and practices into environmentally friendly behavior (Cheema et al., 2020). Indeed, the literature concludes that individuals’ pro-environmental attitude makes them act positively toward nonpolluting activities.

Over the years, national parks have been the prime source of tourist attraction. However, with its growing significance, protecting tourist places has become vital for environmentalists to ensure healthy living. Recently, the employees’ CSR attitude has helped the companies preserve the natural landscape. The individual willingness to initiate and implement the CSR policies highly depends on their attitude toward protecting the earth’s heritage. Given the articulation, the research shows that increasing environmental concerns have made the employees develop a positive attitude toward environment protection activities (Gupta et al., 2021). In particular, the employees’ attitudes and perceptions provide a recreational opportunity, improving the environmental welfare. Tourism is a multi-faced activity that demands the destination’s sustainable development. CSR motivation elevates the employee’s proactive behavior and attitudes (Tian and Robertson, 2019), thus ensuring sustainable tourism development. Employees with a positive environmental attitude respond more responsibly toward the outdoor creational activities. In this regard, the employee attitude is a critical factor that ensures sustainable tourism development (Harun et al., 2018).

Notably, the national park goal identification provides a deeper understanding of protecting the tourism landscape. This information effectively shapes the employees’ attitude toward the most valuable component of the world (i.e., the environment). The CSR attitude manifests the individual behavior toward environmental preservation. Based on this statement, the study shows that the employees’ behavior toward outdoor activities is the outcome of their positive CSR intention (Lin and Lee, 2020). Therefore, this massive engagement in CSR makes the managers and employees develop a favorable attitude toward enhancing the environmental infrastructure of the local tourism destinations. Consequently, based on the previous literature, we concluded the following hypotheses:

*H4: Attitude toward environmental CSR positively and significantly impacts sustainable tourism development.*
*H5: Attitude toward environmental CSR has a positive and significant impact on employee Pro-environmental behavior.*
*H6: Attitude toward environmental CSR has a positive and significant impact on national park goal identification.*

### The Mediating Role of National Park Goal Identification

The national connection to the world’s heritage (i.e., national parks) significantly strengthens the beauty of our planet. As a result, this dominant notion in tourism calls for protecting the natural habitats for healthy living. In the tourism industry, the national parks have formed a critical component influencing the country’s socio-ecological welfare. Concerning its increasing significance, the research suggests that national parks affect the country’s economy while making it essential for meteorologists to focus on its natural features for achieving sustainable tourism development (Stemberk et al., 2018).

Perhaps, over the years, the nation’s destinations have always been the center of tourist attractions. The broad view of the tourism industry enables individuals to form a deep connection with the world’s natural heritage, thus boosting environmental sustainability practices. However, this facilitation encourages the firms to protect the motherland with proper management. This understanding leads the organizations to support tourism infrastructure with sustainable activities. Therefore, to preserve the national legacy, individuals’ (e.g., residents, employees, and organizations) have strived to promote tourism’s sustainability (Olya et al., 2019).

The tourism sector demands a sustainable model that focuses on environmental protection (Higgins-Desbiolles et al., 2019). Perhaps, securing the tourism sector (i.e., national parks) has become an increasing trend worldwide. As a result, the countries and organizations are protecting the areas with maximum legacy. In particular, today, the cleaner environmental perception has made the global industries (e.g., tourism) evolve into a sustainability paradigm. This extended declaration has made individuals adopt sustainability practices, thus improving the employees’ CSR perceptions regarding environmental protection (Pérez-Calderón et al., 2020). The employees’ CSR perception elevates the need for motherland protection. This environmental protection requires the employees to utilize and coordinate the country’s natural resources (Pérez and del Bosque, 2017). As a result, the literature suggests that organizations should develop positive CSR to secure a natural legacy for future generations (Saviano et al., 2018).

However, the literature indicates that for ensuring the sustainability domains (i.e., economic, social, and ecological), the individuals’ have changed their attitudes in response to the increasing environmental deterioration. In particular, the CSR attitude alludes to a psychological tendency that causes the individual to behave favorably toward the environment. Accordingly, in recent years, the literature suggests that the spreading of ecological awareness and CSR knowledge has encouraged the employees’ to balance the firms’ progress with sustainable development (Ahmed et al., 2020). Indeed, today, the current ecological widespread has encouraged the employees ‘to show interest in the environmental welfare. The growing environmental concerns have made the CSR phenomenon preserve the tourist areas. CSR activities influence individuals’ attitudes and behavior (Khattak et al., 2021). In particular, connecting to this concept, the literature focuses on the employees’ CSR attitude toward achieving sustainable tourism development. Employees’ positive CSR perception influences their attitude supporting tourism development. Indeed, the study shows that individuals’ attitudes are the most dominant element in establishing tourism sustainability (Esfandiar et al., 2019). The CSR attitude plays a vital role in enhancing the employees’ perspective toward sustainable development. The positive CSR attitude makes employees responsible, thus significantly contributing to sustainable tourism development (De Roeck and Farooq, 2018). Consequently, the prior research findings states:

*H7: National Park Goal Identification has a positive and significant impact on sustainable tourism development.*
*H7(a): National Park Goal Identification mediates the relationship between environmental CSR perception and sustainable tourism development.*
*H7(b): Attitude toward environmental CSR mediates the relationship between environmental CSR perception and sustainable tourism development.*

### The Mediating Role of Employee Pro-environmental Behavior

The worldwide ecological view has increased individuals’ tendency to engage in eco-friendly behavior, thereby minimizing the undesirable consequences of climate change (Truelove and Gillis, 2018). In an endeavor to explicitly exhibit the employee’s environmentally friendly behavior, the organization has encouraged the individuals to adopt pollutant-free activities to mitigate environmental adversity. Given the illustration, the study shows that the employee pro-environmental behavior ensures environment protection, thus leading to sustainable tourism development (Halder et al., 2020). Hence, the environmental psychology literature suggests adopting eco-friendly behavior to ensure tourism sustainability (Han, 2020).

However, in the last few years, the CSR concept has become a significant aspect, manifesting the individuals’ ecological behavior (Geiger et al., 2019). The employees’ environmental responsibility makes them realize their ecological obligations, thus exhibiting eco-friendly behavior. Indeed, the changing climatic conditions motivate the employees to engage in pro-environmental activities, thus supporting sustainability practices. In the tourism sector, the CSR belief guides the employees’ to exhibit positive environmental behavior, thereby gaining sustainability development (Kiatkawsin and Han, 2017). Therefore, organizations should adopt CSR practices to promote employee pro-environment behavior.

In particular, the effectiveness of the CSR practices elevates the employee’s pro-environmental behavior. The environmental behavior reduces the environmental negativity, thereby making eco-friendly practices promote the ecological behaviors (Quér et al., 2020). Interestingly, the employees’ responsibility toward the ecological behavior requires the future generation to be aware of the environmental standards leading to sustainable development. Accordingly, the study shows that the concept of CSR has massively harmonized the need for nature’s protection against the increasing climatic disruption by taking sustainable environmental actions (Agus Harjoto and Salas, 2017). Indeed, the literature concludes that ecological behavior against environmental activities triggers sustainable outcomes.

Undoubtedly, the escalating climate changes have made the attributes of human-environmental behavior to become the most dominant topic ensuring environmental sustainability. However, today, global environmental problems have demanded the need for eco-friendly behavior in the tourism sector. In explanting this notion, the study states that employees’ pro-environmental behavior brings numerous benefits to the firms, thereby altering the employees’ attitudes toward sustainability (Shah et al., 2021).

Indeed, the pro-environment advocacy has significantly called for nature-based tourism, subsequently promoting environmental sustainability.

Similarly, another research investigating the pro-environmental behavior states that the employees’ positive attitude influences their ecological behavior, thereby enhancing their commitment toward sustainable development (Yusliza et al., 2020). Therefore, the literature suggests that employees’ pro-environmental behavior safeguards the nation’s landscape by significantly altering their attitude toward sustainable practices (Zhang et al., 2021). Notably, considering the CSR activities from the environmental perspective led the stakeholders to direct their attitude toward promoting employees’ pro-environmental behavior. In recent years, the biospheric value orientation has significantly encouraged the employees to exhibit pro-environmental behavior for nature’s protection. Given the articulation, the study shows that recent climatic changes have driven the employees’ attitude toward achieving sustainable development via adopting CSR initiatives (Elf et al., 2021). Hence, to achieve the sustainability goals, individuals should positively perceive the CSR activities to achieve sustainable tourism development. Altogether, the hypotheses conclude:

*H8: Employee pro-environmental behavior has a positive and significant impact on sustainable tourism development.*
*H8(a): Pro-environmental behavior mediates the relationship between environmental CSR perception and sustainable tourism development.*
*H8(b): Pro-environmental behavior mediates the relationship between attitude toward CSR and sustainable tourism development.*

**FIGURE 1:**
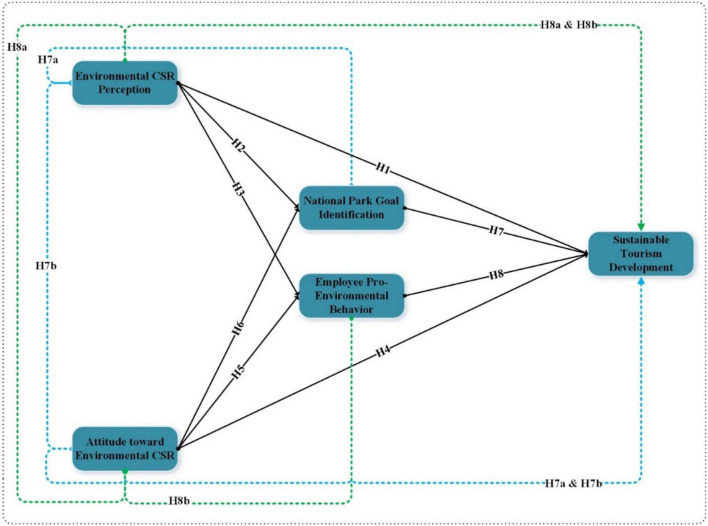
Study conceptual framework.

The study’s independent variables (environmental CSR perception and attitude toward environmental CSR), mediating variables (National Park goal identification and employee pro-environmental behavior), and dependent variable (sustainable tourism development) are shown in Figure 1.

## Methodology

The study aimed to analyze the green innovation behavior toward sustainable tourism development. The data was collected through questionnaires from the tourism firms’ employees located in the different areas of China. The online questionnaires (Google Form) were distributed among 440 employees from November 2021 to March 2022. Three hundred thirty-eight valid questionnaires were received for the data analysis. A statistical package for the social sciences and structural equation modeling (SEM) was used for data analysis and hypothesis testing.

### Measurement Scale

This study has adopted previously developed questionnaire items, and these items were measured on the 5-item Likert scale ranging from (Strongly disagree to Strongly agree). Environmental CSR perception was measured on the 4-items scale developed by Zhang et al. (2021). The sample items include “Our firm participates in the activities which aim to protect and improve the quality of the natural environment” and “Our firm implements special programs to minimize its negative impact on the natural environment.” Attitude toward environmental CSR was measured on the 5-items scale adopted from the study of Zhang et al. (2021). The sample items include “Environmental responsibility is critical to the survival of a business enterprise” and “The overall effectiveness of a business can be determined to a great extent by the degree to which it is environmental responsible.” National Park goal identification was measured on the 4-items scale developed by Zhang et al. (2021). The sample items include “National Park is an ideal model of protecting ecosystem and natural and cultural resources” and “The objective of setting up national parks is to protect natural and cultural resources and ecological environment.” Employee Pro-environmental behavior was measured on the 6-items scale adopted from the study of Fatoki (2019). Sustainable tourism development was measured on the 6-items scale adopted from the study of Elshaer et al. (2021).

## Results

Table 1 shows the study’s descriptive analysis results. These results reveal the frequencies in terms of gender, age group, education, and academic status of the participants of this study. The data was collected from the 338 employees, including 195 male and 193 female participants. Most of the study participants were between 31 and 40 years (27.6%), and 142 participants had a bachelor’s degree.

**TABLE 1:** Demographic characteristics.

Items	Frequency (*N* = 388)	(%)
***Gender***		
Male	195	50.3
Female	193	49.7
***Age***		
19–30	49	12.6
31–40	107	27.6
41–50	100	25.8
51–60	85	21.9
>60	47	12.1
***Education***		
Intermediate	70	18
Bachelor	142	36.6
Master	114	29.4
M.Phil./Others	62	16
***Marital Status***		
Single	192	49.5
Married	196	50.5

### Common Method Bias

This research also applied the common method bias using Harman’s single-factor approach. The variance extracted by one single factor is 10.437% which is less than 50%, indicating that there is no common method bias in this study (Podsakoff et al., 2003).

### Assessment of Measurement Model

The study variable’s reliability and validity analysis were conducted before hypothesis testing. Table 2 factor loadings of each variable for each item. All the construct values were above 0.6, as recommended by Hair (2010) which ranged from 0.680 (EPEB_5) to 0.796 (STD_2), and the average variance extracted (AVE) score was above the cut-off value of 0.50 (Nunnally and Bernstein, 1994). According to Bagozzi and Yi (1988), the CR value should be higher than 0.6. All the study variables’ CR value is within range in this study.

**TABLE 2:** Reliability and validity analysis.

Construct	Items	Loading	α	CR	AVE
Environmental CSR perception	ECP_1	0.723	0.824	0.824	0.540
	ECP_2	0.748			
	ECP_3	0.736			
	ECP_4	0.731			
Attitude toward environmental CSR	AEC_1	0.773	0.861	0.860	0.552
	AEC_2	0.714			
	AEC_3	0.767			
	AEC_4	0.712			
	AEC_5	0.748			
National Park goal identification	NPGI_1	0.739	0.829	0.829	0.548
	NPGI_2	0.710			
	NPGI_3	0.717			
	NPGI_4	0.792			
Employee pro-environmental behavior	EPEB_1	0.724	0.872	0.872	0.533
	EPEB_2	0.734			
	EPEB_3	0.748			
	EPEB_4	0.755			
	EPEB_5	0.680			
	EPEB_6	0.736			
Sustainable tourism development	STD_1	0.727	0.877	0.877	0.543
	STD_2	0.796			
	STD_3	0.689			
	STD_4	0.751			
	STD_5	0.719			
	STD_6	0.734			

Table 3 shows the discriminant validity analysis. The analysis was based on the Fornell-Larcker and HTMT method. Moreover, a more recent method known as Heterotrait-monotrait (HTMT) ratio is also used as a more robust approach to measuring the discriminant validity of the measures.

**TABLE 3:** Discriminant validity analysis (Fornell-Larcker and HTMT).

Constructs	1	2	3	4	5
1. Attitude toward environmental CSR	*0.743*	0.619	0.684	0.675	0.687
2. Environmental CSR perception	0.618	*0.735*	0.646	0.640	0.677
3. Employee pro-environmental behavior	0.685	0.646	*0.730*	0.660	0.676
4. National Park goal identification	0.677	0.641	0.661	*0.740*	0.665
5. Sustainable tourism development	0.687	0.677	0.677	0.666	*0.737*

*Values on the diagonal (italicized) represent the square root of the average variance extracted, while the off diagonals are correlations.*

Table 4 shows variance influence factor values of attitude toward environmental CSR, environmental CSR perception, employee pro-environmental behavior, national park goal identification, and sustainable tourism development. The values were below 3.3, so the current study model is free of collinearity and common method bias (Kock, 2015).

**TABLE 4:** Variance influence factor.

Constructs	1	2	3	4	5
1. Attitude toward environmental CSR			1.619	1.619	2.352
2. Environmental CSR perception			1.619	1.619	2.071
3. Employee pro-environmental behavior					2.375
4. National Park goal identification					2.320
5. Sustainable tourism development					

**FIGURE 2:**
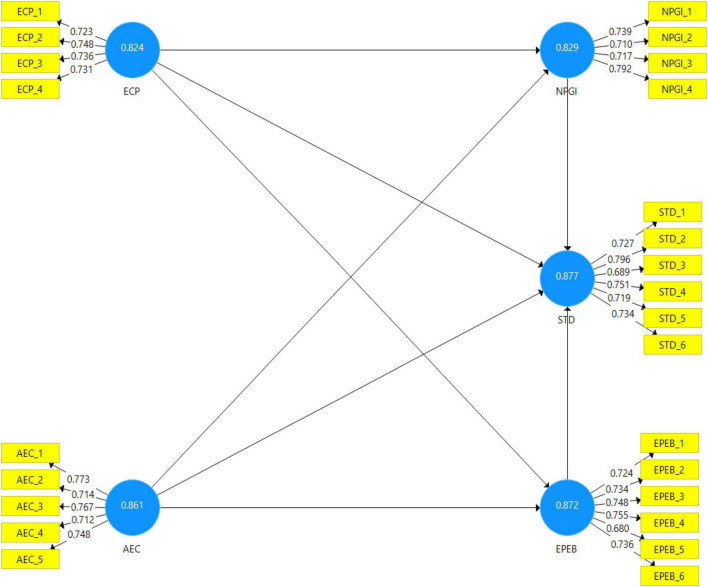
Graphical representation of assessment of measurement model.

Figure 2 shows the graphical representation of the assessment of the measurement model (Independent, dependent, moderating, and mediating variable).

### Structural Model

Table 5 shows the direct hypothesis relationship between study variables. The hypothesis H1–H8 was accepted because the *p*-value was less than 0.05. Hypothesis H1 states that environmental CSR perception has a positive and significant impact on sustainable tourism development; as shown in Table 5, the *t*-value and *p*-value of environmental CSR perception in predicting sustainable tourism development were 4.466 and less than 0.05, respectively. Hypothesis H2 stated environmental CSR perception has a positive and significant impact on national park goal identification. The hypothesis H2 was accepted at the *t*-value of 4.741 and a *p*-value less than 0.05. Hypothesis H3 stated that environmental CSR perception has a positive and significant impact on employee pro-environmental behavior. The hypothesis H2 was accepted at the *t*-value of 4.962 and a *p*-value less than 0.05.

**TABLE 5:** Hypotheses testing direct effect.

Hypothesis	Direct relationships	Std. *beta*	Std. error	*T*-values	*P*-values
H1	ECP ➜ STD	0.268	0.06	4.466	***
H2	ECP ➜ NPGI	0.36	0.076	4.741	***
H3	ECP ➜ EPEB	0.36	0.073	4.962	***
H4	AEC ➜ STD	0.255	0.078	3.286	**
H5	AEC ➜ EPEB	0.462	0.069	6.738	***
H6	AEC ➜ NPGI	0.454	0.073	6.191	***
H7	NPGI ➜ STD	0.185	0.072	2.575	*
H8	EPEB ➜ STD	0.207	0.079	2.631	**

*Indicates significant paths: *p < 0.05, **p < 0.01, ***p < 0.001.

Similarly, hypothesis H4 was accepted at the *t*-value of 3.286 and a *p*-value less than 0.05. The hypothesis (H5) attitude toward environmental CSR has a positive and significant impact on employee Pro-environmental behavior, and hypothesis (H6) attitude toward environmental CSR has a positive and significant impact on national park goal identification were accepted at a *p*-value less than 0.05. The hypothesis H7 and H8 were accepted at the *t*-value of 2.575 and 2.631, respectively.

Table 6 shows the results of mediating variables. All the mediating hypotheses were accepted at a *p*-value less than 0.05. NPGI mediated ECP ➜ STD (β = 0.067*, *t* = 2.119). Hypothesis H7(b) stated that NPGI mediated AEC ➜ STD (β = 0.084, *t* = 2.303). Hypothesis H8(a) shows that pro-environmental behavior mediates the relationship between environmental CSR perception and sustainable tourism development. Bootstrapping results show that pro-environmental behavior mediated environmental CSR perception and sustainable tourism development at the beta value of 0.075. Hypothesis H8(b) shows that pro-environmental behavior mediates the relationship between attitude toward CSR 302 sustainable tourism development. Bootstrapping results show that pro-environmental behavior mediated attitudes toward CSR and sustainable tourism development. at the beta value 0.096 (AEC ➜ EPEB ➜ STD).

**TABLE 6:** Hypotheses testing mediation effect.

Hypothesis	Indirect relationships	Std. *beta*	Std. error	*T*-values	*P*-values
H7a	ECP ➜ NPGI ➜ STD	0.067	0.031	2.119	*
H7b	AEC ➜ NPGI ➜ STD	0.084	0.036	2.303	*
H8a	ECP ➜ EPEB ➜ STD	0.075	0.036	2.091	*
H8b	AEC ➜ EPEB ➜ STD	0.096	0.041	2.353	*

*Indicates significant paths: *p < 0.05.

**FIGURE 3:**
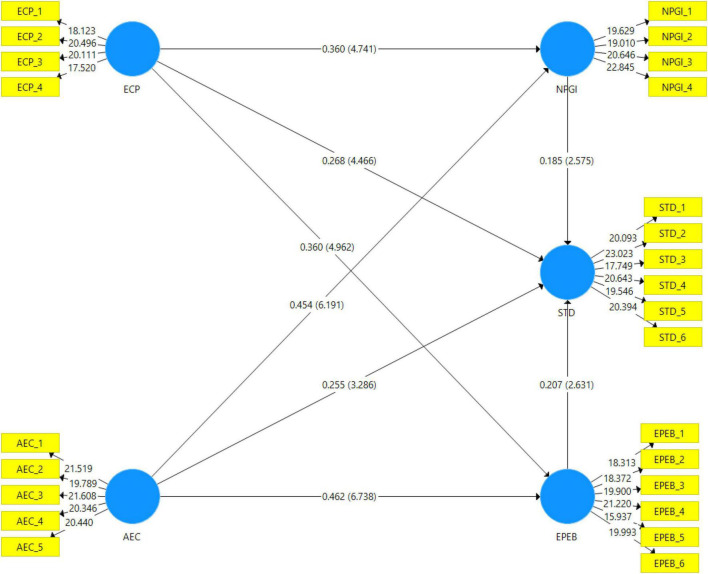
Graphical representation of the structural model.

Figure 3 is a graphical representation of the structural model.

## Discussion

In particular, section “Discussion” sheds light on the different notions of tourism development. This discussion section provides an in-depth review of the study results in correspondence to the previous literature reviews. Accordingly, the prior literature infers that CSR perception and sustainable tourism development have gained employees’ attention. In recent years, the adoption of CSR has strengthened the employees’ commitment to sustainable development. In particular, environmental CSR is a vital component that creates an influential impact on tourism development. In the illustration, the study states that CSR enhances the individuals’ corporate ecological responsibility, thereby fostering the organization’s environmental development (Gond et al., 2017). In the same vein, the study results had found consistent with the previous literature that has also recorded a significant relationship between CSR perception and sustainable tourism development. Indeed, these current findings made us support and accept the H1.

Moreover, the increasing CSR has made the firms more concerned about natural landscape preservation. The CSR approach ensures the protection of biodiversity. The positive consequence of CSR has made the organizations support the conversation movement for the livelihood of the national parks. In explaining this notion, the study states that the increasing CSR awareness has made the employees respond to the anthropogenic pressure, thereby encouraging taking initiatives for the motherland protection (Marasinghe et al., 2021). In particular, the prior literature also supports the study outcome by making us accept the H2. In the same vein, the previous studies explain that the CSR perception triggers the employees’ protection behavior. The positive CSR efforts make the employees respond to environmental protection through developing positive ecological behavior. Ecologically sustainable behavior benefits the entire society by translating the workers’ pro-environmental advocacy to ensure societal well-being (Alsuwaidi et al., 2022). However, this literature supports and accepts our findings regarding CSR perception and employee pro-environmental behavior (i.e., H3).

Similarly, the literature shows that the employees’ environmental attitude profoundly escalates the environmentally responsible behavior to enhance the beauty of the world’s heritage sites. Natural destination protection shapes the employees’ pro-environmental actions, thus facilitating tourism development (Watto et al., 2020). Therefore, the prior study confirms a positive relationship between CSR attitude, national park identification goals, and sustainable development, fundamentally supporting H4, H5, and H6. Moreover, in understanding the mediating role of the national park identification goal, the study shows that sustainable tourism development has enhanced the understanding of ecological conservation (Hsu, 2019). Employee protective actions encourage the firms to protect the potential motherland. Natural heritage protection requires the employees’ to preserve the world’s natural area (Ristić et al., 2019). In particular, the literature states that developing a sustainable park requires employees to practice CSR activities, thus protecting the nation’s heritage (Jamaliah et al., 2019). Indeed, all these prior studies support the current study results (H7, H7a, and H7b).

Notably, the tourism sector demands the employees to secure the tourists’ destinations. The employees’ pro-environmental behavior contributes toward sustainable tourism where the CSR practices foster the environmental well-being. In particular, today, numerous emerging markets have become attractive to the ecological beauty of the tourism areas that have suffered colossally due to the increasing environmental degradation. The employee’s pro-environmental behavior addresses the environmental concerns by enhancing outdoor recreational activities (Liu et al., 2021). Based on this statement, the study reveals that CSR plays an integral role in improving the employees’ pro-environmental behavior (Ogbonnaya and Messersmith, 2019). Therefore, the prior literature supports the current study findings by confirming a significant mediating role of pro-environmental behavior in achieving sustainable tourism development (e.g., H8, H8a, and H8b). Overall, the study findings record a positive relationship between all the variables. All the results had found significant, thereby fundamentally supporting all the proposed hypotheses.

The current study also offers some limitations. First, the data was collected only from the Chinese tourist firms. Future studies can be cross-national, and data can be collected from different countries. It will provide a better understanding of sustainable tourism development. Secondly, the current study has considered mediating variables, so future studies might consider some organization-level moderating variables for testing the same model.

## Conclusion

Over the years, the world’s meteorologists have directed the organizations’ attention toward the world’s environmental welfare. Today, the increasing value of sustainable development and heritage protection has made organizations protect the world’s heritage sites. Previously, CSR studies have highlighted the need for stakeholders’ participation in improving ecological welfare. But today, the studies have shown that the employees’ CSR perceptive and pro-environmental behavior plays a critical role in ensuring the organization’s survival and sustainable tourism development. Indeed, this current study investigates the role of corporate social responsibility by examining the mediating role of national park identification goals and employee pro-environmental behavior in achieving sustainable tourism development. Environmental CSR perception and attitude toward environmental CSR is significantly related to sustainable tourism development.

## Data Availability Statement

The raw data supporting the conclusions of this article will be made available by the authors, without undue reservation.

## Ethics Statement

Ethical review and approval was not required for the study on human participants in accordance with the Local Legislation and Institutional Requirements. The patients/participants provided their written informed consent to participate in this study.

## Author Contributions

The author confirms being the sole contributor of this work and has approved it for publication.

## Conflict of Interest

The author declares that the research was conducted in the absence of any commercial or financial relationships that could be construed as a potential conflict of interest.

## Publisher’s Note

All claims expressed in this article are solely those of the authors and do not necessarily represent those of their affiliated organizations, or those of the publisher, the editors and the reviewers. Any product that may be evaluated in this article, or claim that may be made by its manufacturer, is not guaranteed or endorsed by the publisher.
